# On the Formability of Medium Mn Steel Treated with Varied Thermal Processing Routes

**DOI:** 10.3390/ma16010258

**Published:** 2022-12-27

**Authors:** Baolin Zhang, Binbin He

**Affiliations:** Department of Mechanical and Energy Engineering, Southern University of Science and Technology, Shenzhen 518055, China

**Keywords:** formability, ductility, intercritical annealing, quenching and partitioning, medium Mn steel

## Abstract

In this contribution, we investigate the influence of thermal processing routes on the formability of medium Mn steel by assessing the strain hardening coefficient and anisotropy factor using the uniaxial tensile test. Medium Mn steel processed by intercritical annealing (IA) at 680 °C for 4 h demonstrates better formability than steel treated with a combination of IA at 800 °C for 10 min and quenching and partitioning (Q&P), based on the much higher strain hardening coefficient (*n*) and comparable anisotropy factor (*r*, *r_m_*, ∆*r*). The higher strain hardening coefficient of medium Mn steel with single IA treatment is ascribed to the enhanced transformation-induced plasticity (TRIP) effect resulting from the large amount of austenite that is transformed into martensite during deformation. In addition, the IA process allows for the production of medium Mn steel with high ductility, which is beneficial for its high formability and good ‘part ductility’ in lightweight automotive applications.

## 1. Introduction

The development of advanced high-strength steels (AHSS) dates back to the early 1980s, when it was developed with the purpose of resolving the conflict between the weight reduction and passenger safety in the automotive industry [1]. Its high strength allows for the use of thin structural components, and can thereby reduce vehicle weight while maintaining the high capability in terms of passenger protection during crash events [1,2]. The manufacturing of body-in-white automobiles based on AHSS can be realized by either the hot stamping or cold stamping techniques. Press-hardening steel (PHS) such as 22MnB5 is particularly designed to cater to the hot stamping technique, where the structural components are made at the austenite regime followed by die quenching to generate lath martensite, resulting in high strength of 1500 MPa and tensile ductility of less than 8% [3]. Unlike the hot stamping technique, the cold stamping process is carried out on AHSS at the room temperature without additional heating, which reduces greenhouse gas emissions during the manufacturing process. The extent of cold stamping in forming complex structural components relies on the formability of AHSS; the formability is used to describe the resistance of sheet metals to strain localization and fracture under complex stress/strain conditions [4,5].

The formability of steel can be evaluated by considering different parameters, including the anisotropy ratio, total/uniform elongation, edge sensitivity, internal stress, and strain rate sensitivity [6]. There are several different testing methods used to estimate the formability, including the square-cup deep-drawing test, the Olson cup test, and the limiting dome height (LDH) test [7,8]. However, no single mechanical test can provide an effective evaluation of the formability of newly developed AHSS (i.e., medium Mn steel). The square-cup deep-drawing test has been used to measure the formability of steel under complex forming conditions, which can be assisted by combining experiments and simulations [9,10]. Such testing has been adopted to investigate the problems associated with the production of complex shapes during sheet hydro-forming processes using a numerical approach [11]. A formability comparison between medium Mn steel and high Mn steel has been carried out using the same tests [12], leading to the conclusion that medium Mn steel has a homogeneous martensitic microstructure after deformation, and demonstrates better formability as a result. In addition to the square-cup deep-drawing test, the uniaxial tensile test is widely used to gauge the formability of steels. The local and global formability of medium Mn steel has been investigated by considering the forming limit curve at fracture [13], suggesting that the uniaxial tensile properties can provide insight into the formability of sheet metals. The formability of medium Mn steel with ultrafine dual-phase microstructure containing ferrite and austenite grains is greatly influenced by the martensitic transformation during the forming process [14].

Most of the existing studies on the formability of medium Mn steel focus on steel processed using the single intercritical annealing (IA) process [13,15,16,17]. The formability of medium Mn steel developed by newly designed thermal processing routes such as intercritical annealing combined with a quenching and partitioning (IA-Q&P) process has not yet been studied. Medium Mn steel with 5 Mn wt.% processed by IA generally contains a dual-phase ultra-fine-grained microstructure [18]. The soft ferrite matrix leads to relatively low yield strength. With increasing Mn content up to 10 wt.%, a larger volume fraction of retained austenite can be obtained after IA. In contrast, medium Mn steel processed by IA-Q&P incorporates a triple phase including ferrite, retained austenite, and martensite. The introduction of martensite can effectively improve the yield strength. The prior austenite grain size can be refined through the IA process, which leads to the formation of fine martensite laths. The formation of small martensite is important in optimizing the mechanical stability of retained austenite grains by tuning their grain size and morphology [19]. Therefore, considering the different microstructures and mechanical properties resulting from varied processing routes, the present work investigates the formability of medium Mn steel processed by different thermal processing routes, including the IA and IA-Q&P processes [20]. Based on the strain hardening coefficient and plastic strain ratio obtained from systematic uniaxial tensile tests along different directions, in this paper we thoroughly discuss which thermal processing route is more desirable in achieving high formability for lightweight automotive applications.

## 2. Experiments

**Figure 1:**
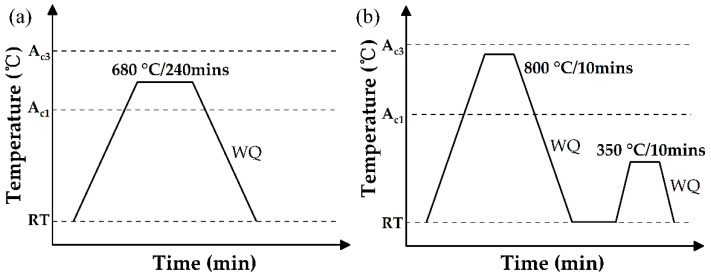
Schematic illustration of thermal processing routes employed in the present work, including the (**a**) IA and (**b**) IA-Q&P processes. WQ: water quenching; RT: room temperature.

The steel employed in the present investigation had a nominal chemical composition of Fe-10Mn-0.2C-2Al-0.1V (in wt.%). This alloy composition was used to develop room temperature quenching and partitioning (RT Q&P) steel in our previous studies [20,21,22]. An amount of this steel weighing 50 kg was prepared by conventional casting into an ingot, followed by forging to obtain a steel plate with a thickness of 12 mm and a width of 100 mm. The forged steel was homogenized at 1150 °C for 2 h to dissolve the vanadium carbide, then hot rolled down to 4 mm in thickness and air cooled to room temperature. An isothermal holding at 680°C for 10 h was performed to soften the hot rolled plate in order to realize a thickness reduction of 50% for subsequent cold rolling. Sub-standard tensile specimens with a gauge dimension of 25 mm × 6 mm × 2 mm were prepared according to ASTM-E8 along the different directions using wire electrical discharge machining. In particular, the tensile specimens were machined along the rolling direction (RD), transverse direction (TD), and at 45° to both RD and TD, which are hereafter referred to as the 0°, 90°, and 45° samples, respectively. The tensile samples were prepared from the same steel plate in order to avoid the possibility of heterogeneity across different plates. The tensile specimens were subjected to two different heat treatments (Figure 1): intercritical annealing at 680 °C for 240 min followed by subsequent water quenching; and intercritical annealing at 800 °C for 10 min, quenching in water, and partitioning at 350 °C for 10 min followed by final quenching in water to room temperature. The higher intercritical annealing temperature leads to a higher volume fraction of austenite, and thus to lower mechanical stability of the austenite grains (less C/Mn partitioning from the ferrite grains and possibly larger austenite grain size). Thus, the austenite grains after intercritical annealing at 680 °C are already stable enough to resist the martensitic transformation during quenching to room temperature. In contrast, the austenite grains after intercritical annealing at 800 °C are not stable, and the martensitic transformation takes place during water quenching, which is the basis for the application of the IA-Q&P processing route. The rationale for selecting the present thermal processing routes can be found in our previous study [20]. The corresponding steels are hereinafter termed IA steel and IA-Q&P steel for brevity. Note that the isothermal holding at 680 °C and 800 °C is within the dual-phase regime, which is beneficial in controlling the austenite grain size [23]. The tensile test was carried out using the universal tensile machine (Hualong, Shanghai, China) under a cross-head speed of 1.2 mm/min at room temperature. The initial and deformed microstructure of the steel treated with different thermal processing routes was characterized by scanning electron microscopy (SEM) using a Merlin Field Emission SEM (Zeiss, Oberkochen, Baden-Wuerttemberg, Germany). The sample for SEM observation was prepared by mechanical polishing followed by electrolytic polishing in a mixed solution of ethanol and perchloric acid at a ratio of 9:1 (vol.%), a voltage of 18 V, and a current of 900 mA. X-ray diffraction (XRD) tests were performed on the electro-polished samples using a Co Kα radiation source with a wavelength of 1.788 Å in a D8 Advance (Bruker, Billerica, MA, USA). The austenite volume fraction was estimated based on the integrated intensities of the diffraction reflections, including the peaks at (2 0 0)*α*, (2 1 1)*α*, (2 0 0)γ, (2 2 0)*γ*, and (3 1 1)*γ*.

## 3. Results and Discussion

**Figure 2:**
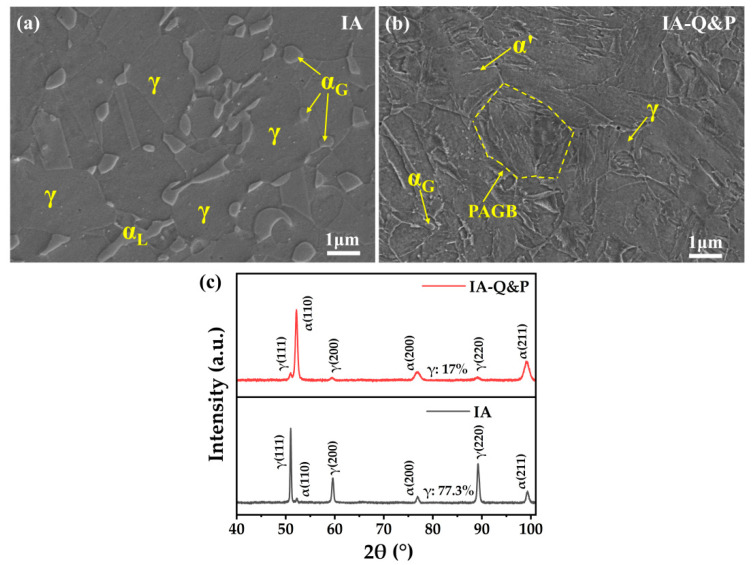
Initial microstructure in SEM (**a**,**b**) and XRD profiles (**c**) of IA steel and IA-Q&P steel. PAGB: prior austenite grain boundary; α_L_: lamellar ferrite; α_G_: granular ferrite; γ: austenite; α’: martensite. XRD profiles showing.

After the intercritical annealing at 680 °C for 4 h, the steel demonstrates a dual-phase microstructure with ultrafine ferrite grains embedded in the austenite matrix (Figure 2a). Both lamellar and granular ferrite grains are observed, with the proportion of granular grains being substantially larger than lamellar ones. The different morphology of ferrite grains could relate to the varied recrystallization state of ferrite grains, as the granular ferrite grains are fully recrystallized [24]. The volume fraction of austenite in the steel treated with IA is 77.3%, according to the XRD measurement. Unlike the microstructure of IA steel, the IA-Q&P steel consists of ultrafine ferrite grains, lath martensite, and retained austenite grains (Figure 2b). The intercritical annealing at 800 °C for 10 min results in a dominated austenitic phase with limited C/Mn enrichment [25], and the austenite grains are not stable enough to resist the martensitic transformation during water quenching to room temperature. Nevertheless, C enrichment of the austenite is realized through the partitioning of C atoms from the transformed martensite during the tempering process [26,27]. According to the XRD profile, the volume fraction of retained austenite in the IA-Q&P steel is 17.0% (Figure 2c). Note that the volume fraction of austenite in IA steel and IA-Q&P steel in the present study is different from that reported in our previous study, which could be due to the different XRD sources (Co/Cu sources, respectively) and the varied microstructures obtained by using different air furnaces (temperature fluctuation).

**Figure 3:**
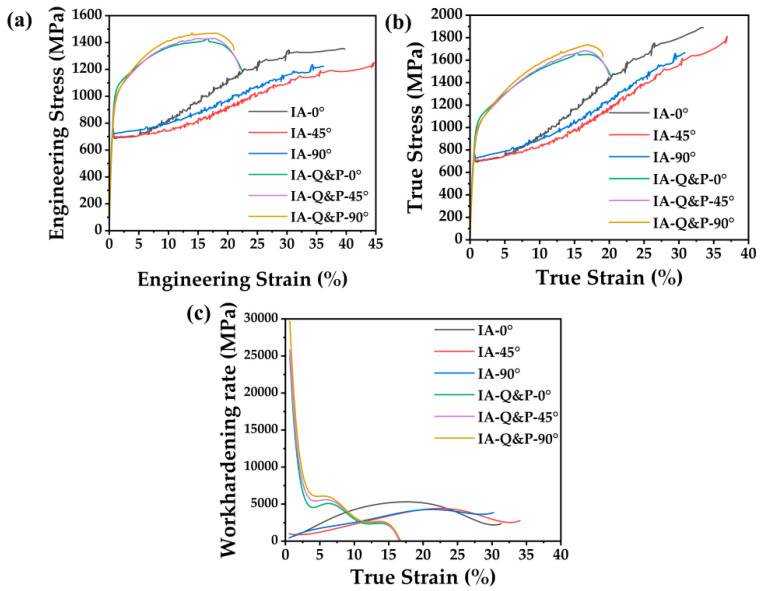
(**a**) Engineering stress–strain curves, (**b**) true stress–strain curves, and (**c**) work hardening rate curves of the steel processed by IA and IA-Q&P heat treatments along the 0°, 45°, and 90° directions with respect to the rolling direction.

The tensile behavior of the steel treated with varied processing routes along the different loading directions is shown in Figure 3a. Note that the tensile properties of the medium steel processed by either IA or IA-Q&P in the present study is different from that reported in our previous study [20], which could be due to the different air furnaces that we used in these two studies. The typical yield drop is observed in the engineering stress–strain curves of the IA steel along the 0°, 45°, 90° directions, followed by the propagation of Lüders band and the subsequent occurrence of the Portevin-Le Chatelier (PLC) band. The jerky stress within PLC band could be due to intermittently localized martensite formation during tensile straining [28]. Such tensile behavior is typical for medium Mn steel treated with a prolonged intercritical annealing process [29,30,31]. The yield strength of IA steel along the 0°, 45°, and 90° directions is 690 MPa, 700 MPa, and 720 MPa, respectively, while the corresponding total elongation is 39.8%, 44.7%, and 36.2%, respectively (Table 1). Note that the operation of the PLC effect and the grinding quality of the surface may lead to the different total elongation values. Unlike the IA steel, continuous yielding followed by strain hardening with a slight PLC effect is observed in the IA-Q&P steel (Figure 3a). The generation of martensite during the quenching process results in the distribution of mobile dislocations in the ferrite and austenite, which contributes to the continuous yielding process [32]. The yield strength of IA-Q&P steel along the 0°, 45°, and 90° directions is 990 MPa, 935 MPa, and 895 MPa, respectively (Table 1). IA-Q&P-0° steel shows similar UTS with IA-Q&P-45° steel, with both values about 50MPa lower than that of IA-Q&P-90° steel. Based on the true stress–strain curves (Figure 3b), the work hardening rate of both IA steel and IA-Q&P steel can be plotted as shown in Figure 3c. The work hardening rate of IA steel can be maintained at a high value over a large strain regime compared to that of IA-Q&P steel (Figure 3c), which could be due to its higher austenite volume fraction making for an enhanced transformation-induced plasticity (TRIP) effect [33,34]. Although a subtle difference in the work hardening behavior of these two steels along different directions can be detected, in the general trend of the work hardening behavior they are coincident with each other (Figure 3c).

**Figure 4:**
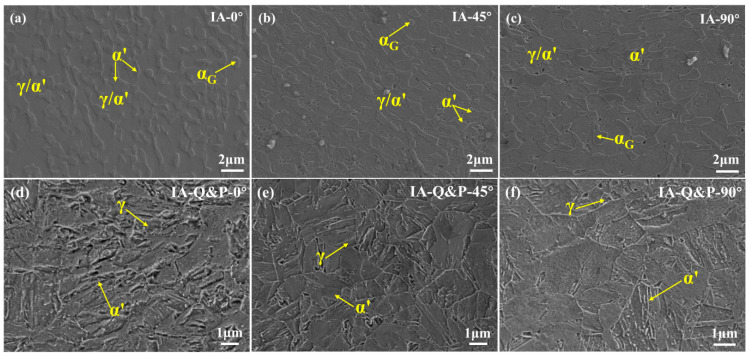
SEM images showing the deformed microstructure of (**a**–**c**) IA steel and (**d**–**f**) IA-Q&P steel after the uniaxial tensile test along the (**a**,**d**) 0°, (**b**,**d**) 45°, and (**c**,**f**) 90° directions with respect to the rolling direction. The microstructure is observed at the uniformly deformed region. α: ferrite, γ: austenite, α’: martensite.

The deformed microstructure of the IA steel and IA-Q&P steel was observed by SEM, with the results shown in Figure 4. Compared to the initial microstructure (Figure 2a), the deformed microstructure of IA steel along the different directions (Figure 4a–c) consists of a more convex phase, which may be due to the formation of martensite during the tensile test. The deformed microstructure of IA-Q&P steel along the different directions (Figure 4d–f) is chaotic compared to the initial microstructure (Figure 2b). It should be noted that the transformed martensite is not clearly etched during electropolishing owing to its having the same carbon content as the surrounding austenite matrix. Thus, the volume fraction of transformed martensite in both the IA and IA-Q&P steel is difficult to obtain through morphological observation.

**Figure 5:**
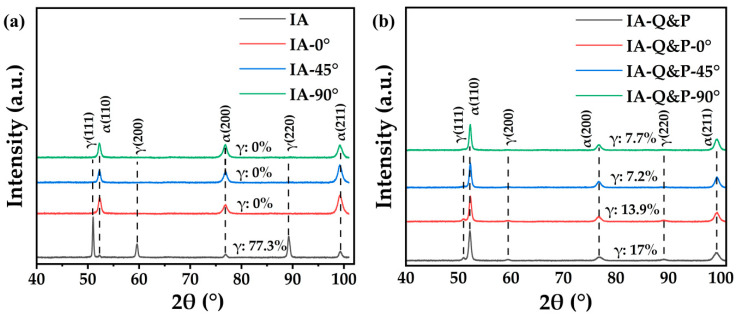
The XRD profiles of (**a**) the IA steel and (**b**) the IA-Q&P steel prior to and after the tensile test along different tensile directions (0°, 45°, 90°). The measurement was performed at the uniformly deformed region.

The volume fraction of austenite was estimated based on the XRD profiles, as shown in Figure 5. The intensity of the austenite peaks decreases after the tensile test along the different directions for both IA steel and IA-Q&P steel, confirming the operation of the TRIP effect in all tested samples. In particular, the austenite peak in IA steel after tensile deformation is not discernible, suggesting that most of the austenite grains are transformed into martensite (Figure 5a). In contrast, a proper amount of austenite grains remain in the IA-Q&P steel after deformation, with 13.9%, 7.2%, and 7.7% the for 0°, 45°, and 90° directions, respectively (Figure 5b). Thus, the transformed amount of austenite in the IA steel is much larger than that of the IA-Q&P steel during tensile deformation (Figure 5), which is consistent with the improved work hardening behavior of IA steel owing to the enhanced TRIP effect, which in turn results from the substantial martensitic transformation with large volume fraction (Figure 3c). The transformed martensite in IA steel across the different directions is similar, which suggests that the overall martensitic transformation is less affected by the loading direction in IA steel (Figure 5a). However, the transformed amount of austenite in IA-Q&P steel along varied tensile directions is slightly different (Figure 5b), which may be due to the presence of certain microstructure textures resulting from partial recrystallization during isothermal holding at 800 °C for a short duration of 10 min. Note that the trend of martensitic transformation in the present medium Mn steel with varied processing routes along the 0° direction is different from the counterparts reported in our previous study [20]. The volume fraction of austenite grains in the IA steel in this study is larger than that in the prior study [20], meaning that the present austenite grains have lower Mn/C enrichment, and thus lower mechanical stability. This may lead to the almost full transformation of austenite into martensite in the current study, while a few residual austenite grains were detected after tensile deformation in the previous study [20]. In contrast, the austenite volume fraction in the IA-Q&P steel was smaller than in the previously [20], which may have led to austenite grains with higher C enrichment and more significant shielding effect from surrounding martensite, and thus higher mechanical stability and a higher volume fraction of austenite remaining after the tensile test. The higher mechanical stability of austenite grains in the IA-Q&P steel as compared to the IA steel in the present study could be due to the finer austenite grain size (Figure 2a,b) and the shielding effect from the strong martensite matrix.

**Figure 6:**
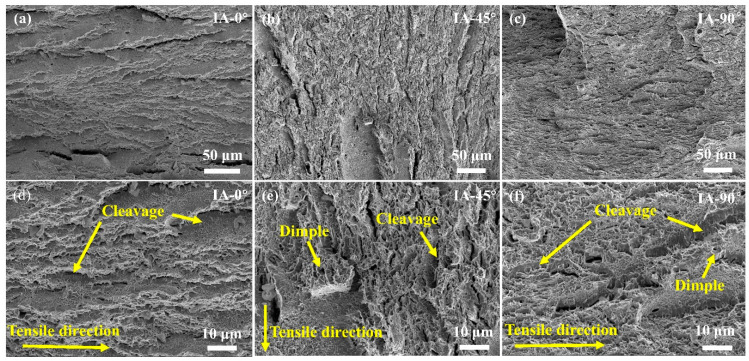
Fracture morphology of IA steel after the uniaxial tensile test along the (**a**,**d**) 0°, (**b**,**e**) 45°, and (**c,f**) 90° directions with respect to the rolling direction; (**d**–**f**) is a magnified view of (**a**–**c**).

The fracture surface of IA steel after the tensile test along the different directions was subjected to SEM observation (Figure 6). Irrespective of the different loading directions, the fracture surface of IA steel is dominated by fine dimples while the cleavage fracture can be occasionally detected, suggesting its excellent tensile toughness (the area beneath the engineering stress-strain curve). The fine dimples could relate to the ultra-fine grain size of the austenite and ferrite (Figure 2a). The large cracks parallel to the tensile direction in IA-0° are observed in the IA-45° as well, and are absent in the IA-90° (Figure 6a–c), which could be due to variations in the observed locations. In general, the above observation is consistent with the similar tensile behaviors of IA steel along the different directions (Figure 3a).

**Figure 7:**
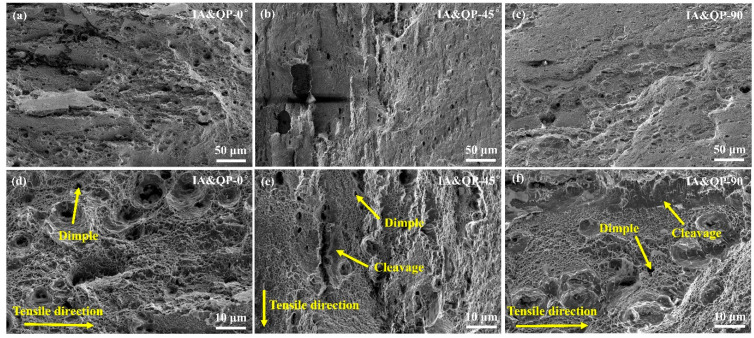
Fracture morphology of the IA-Q&P steel after the uniaxial tensile test along the (**a**,**d**) 0°, (**b**,**e**) 45°, and (**c**,**f**) 90° directions with respect to the rolling direction; (**d**–**f**) is a magnified view of (**a**–**c**).

The fracture surface of the IA-Q&P steel along the different tensile directions is shown in Figure 7. Both dimple and cleavage fractures can be detected on the fracture surface. As compared to the fracture surface of IA steel (Figure 6), the IA-Q&P steel is decorated with a much higher amount of large dimples with a diameter of around 10 μm. These large dimples could be related to the relatively coarse microstructure of the IA-Q&P steel (Figure 2b). Cleavage fractures are distributed more extensively in the IA-Q&P steel as compared to the IA steel (Figure 6 and Figure 7), which is consistent with the shorter total elongation of IA-Q&P steel. Considering the limited martensitic transformation in the retained austenite grains in IA-Q&P steel (Figure 5b), the observed cleavage fracturing could be due to the insufficient tempering of martensite during the partitioning process at 350 °C for 10 min.

The formability of sheet metals can be indicated by the different critical parameters, including the strain hardening coefficient (*n*) and plastic strain ratio (*r*). The higher *n* value indicates the larger ratio of ultimate tensile strength over yield strength, representing better formability [35]. The strain-hardening coefficient of metallic materials is defined by the following equation:

(1) $$n=\frac{d\ln\sigma_{T}}{d\ln\varepsilon_{T}}$$

where *σ_T_* is the true stress and *ε_T_* is the true strain. The *n* value of the IA steel and IA-Q&P steel along different directions can be calculated from the Figure 3b based on equation (1) and shown in Table 2. The *n* value of the IA steel is around 0.52–0.62 for different loading directions, which is much larger than that of IA-Q&P steel (0.19–0.22), suggesting the better formability of IA steel. The higher *n* value of IA steel compared to IA-Q&P steel can be ascribed to its enhanced TRIP effect, in turn owing to its larger fraction of transformed martensite during the tensile test. Nevertheless, both IA steel and IA-Q&P steel demonstrate a much higher *n*-value compared to commercial DP1000 steel with a similar strength level (Table 2). In other words, both IA steel and IA-Q&P steel have higher formability in comparison to the commonly produced DP steel, which is probably due to the TRIP effect resulting from the martensitic transformation from metastable austenite grains in both steels.

In addition to the strain hardening coefficient, the plastic strain ratio (*r*) of the steel is another important parameter in evaluating formability for automotive applications. The *r* value is a measure of the ability of a metallic material to resist thinning during the forming process [36,37]. Note that the *r* value is closely related to the tensile properties, and becomes credible only when the elongation of steel reaches above 15% in the uniaxial tensile test. The plastic strain ratio is known as the anisotropy ratio, and is defined by the following equation:

(2) $$r=\frac{\varepsilon_{w}}{\varepsilon_{t}}=\frac{\ln\left(\frac{w}{w_{0}}\right)}{\ln\left(\frac{t}{t_{0}}\right)}$$

where *ε_w_* is the true strain along the sample width *w* in the uniformly deformed region and *ε_t_* is the true strain along the sample thickness *t*. Considering the complex deformation state during the sheet metal forming process, it is necessary to calculate the average anisotropy (*r_m_*) and planar anisotropy (∆*r*) of tensile tested samples along different directions with respect to the rolling direction. The average anisotropy and the planar anisotropy are defined as follows:

(3) $$r_{m}=\frac{\left(r_{0}{}_{{}^{\circ }}+2r_{45}{}_{{}^{\circ }}+r_{90}{}_{{}^{\circ }}\right)}{4}$$

(4) $$\Delta r=\frac{\left(r_{0}{}_{{}^{\circ }}-2r_{45}{}_{{}^{\circ }}+r_{90}{}_{{}^{\circ }}\right)}{2}$$

where the subscripts 0°, 45°, and 90° refer to the angle between the rolling direction (RD) and the transverse direction (TD). The values of *r*, *r_m_*, and ∆*r* of IA steel and IA-Q&P steel can be calculated based on the tensile tests according to Equations (2)–(4); the results are shown in Table 2. The average anisotropy (*r_m_*) of IA steel is smaller than that of IA-Q&P steel, and is comparable to that of Fe-5Mn-0.1C treated by intercritical annealing (Table 2). The *r_m_* of IA steel is 0.90, which is close to isotropic material, suggesting synchronous deformation along the width and thickness directions. Therefore, the isotropic mechanical properties of IA steel enable better formability, effectively avoiding the formation of cracks and wrinkles during the forming process. The absolute ∆*r* value of IA-Q&P steel is 0.13, which is slightly smaller than that of IA steel (−0.175), though larger than that of Fe-5Mn-0.1C (−0.061). The absolute ∆r value of IA steel is slightly larger than that of IA-Q&P steel, suggesting a relatively higher anisotropy tendency and low likelihood of tearing at the edge of the complex-shaped part during the forming process. Generally, the values of *r_m_* and ∆*r* indicate that both IA and IA-Q&P steel are relatively isotropic and that the influence of the strain path on the mechanical properties is minor. Therefore, based on their average anisotropy and planar anisotropy, both IA steel and IA-Q&P steel demonstrate excellent formability during the forming process.

In evaluating the formability of sheet metals, in addition to the strain hardening coefficient and plastic strain ratio, the total elongation is an important parameter for measuring the applicability of advanced high-strength steels in the cold forming process. The formability gauges the potential of materials to form complex shapes; this capability is termed ‘material ductility’ [6]. After the forming process, structural components should demonstrate sufficient deformation capacity to prevent failures during high energy impacts such as crash events. This capability is known as ‘part ductility’ [6]. Owing to its higher formability, the IA steel demonstrates better ‘material ductility’ compared to the IA-Q&P steel based on the strain hardening coefficient and plastic strain ratio. Moreover, the substantially larger tensile elongation of IA steel (36.2%–44.7%) compared to IA-Q&P steel (21.0–22.4%) means that components made of IA steel would be more deformable compared to counterparts made of IA-Q&P steel. In other words, IA steel enables components with higher ‘part ductility’ than those made of IA-Q&P steel. The IA&QP steel demonstrates humps in the tensile curves at engineering strains larger than 10% (Figure 3a), which may manifest the operation of the PLC effect. However, the intensity of PLC in IA&QP steel is much less severe than in IA steel (Figure 3a). The role of PLC is similar to the Lüders band in affecting the manufacturing process, as both produce surface roughness of the component after forming process without affecting the overall formability. In other words, although the presence of the Lüders band and the more obvious PLC effect in IA steel impairs the surface quality of structural components [38], IA steel can simultaneously secure successful forming while achieving enough residual part ductility owing to its total elongation of over 30%. Consequently, the IA process demonstrates higher potential compared to the IA-Q&P process in meeting the demands of high formability and good ‘part ductility’ for lightweight automotive applications.

## 4. Conclusions

The present work investigates the effect of different thermal processing routes on the formability of medium Mn steel by using the uniaxial tensile test along the different tensile directions. It is found that medium Mn steel processed by IA has a much higher strain hardening coefficient (*n*) and comparable anisotropy factor (*r*, *r_m_*, ∆*r*) compared to steel treated with IA-Q&P, indicating that the IA process is more beneficial than the IA-Q&P process in terms of formability. The higher strain hardening coefficient of IA steel is due to its enhanced TRIP effect resulting from the large austenite volume fraction. Irrespective of the surface quality of structural components, the IA process is more favorable compared to the IA-Q&P process in resolving the demands of high formability and good ‘part ductility’ for lightweight automotive applications.

## Figures and Tables

**Table 1:** Mechanical properties of the IA steel and IA-Q&P steel along three different directions (0°, 45°, 90°).

	Steels	IA			IA-Q&P		
Properties		0°	45°	90°	0°	45°	90°
YS (MPa)		690	700	720	990	935	895
UTS (MPa)		1350	1250	1235	1425	1430	1470
TE (%)		39.8	44.7	36.2	22.4	22.0	21.0

**Table 2:** The *n*, *r*, *r_m_*, and ∆*r* values of IA and IA-Q&P steel compared to other high-strength steels. (“-“ means no value in reference.).

Sample		*n*	*r*	*r_m_*	∆ *r*
IA	0°	0.62	0.64	0.90	−0.175
	45°	0.57	1.08		
	90°	0.52	0.82		
IA-Q&P	0°	0.19	0.92	0.79	0.13
	45°	0.21	0.66		
	90°	0.22	0.93		
Fe-5Mn-0.1C [15]	0°	-	0.847	0.90	−0.061
	45°	-	0.93		
	90°	-	0.891		
DP1000 [13]		0.075	-	-	-

## Data Availability

The raw data required to reproduce these findings are available from the corresponding author of this paper.
